# The presence of human respiratory syncytial virus in the cerebrospinal fluid of a child with Anti-N-methyl-D-aspartate receptor encephalitis of unknown trigger

**DOI:** 10.1186/s12985-023-01997-1

**Published:** 2023-02-24

**Authors:** Siyan Yu, Ying Hua, Jun Qian, Mingxia Sun, Yan-Jun Kang

**Affiliations:** 1 Pediatric Laboratory, Wuxi Children’s Hospital Affiliated to Nanjing Medical University, Wuxi, Jiangsu China; 2 The First School of Clinical Medicine, Nanjing Medical University, Nanjing, Jiangsu China; 3 Department of Neurology, Wuxi Children’s Hospital Affiliated to Nanjing Medical University, Wuxi, Jiangsu China

**Keywords:** Anti-N-methyl-D-aspartate receptor encephalitis, Human respiratory syncytial virus, Children, Transcriptome sequencing

## Abstract

**Background:**

Anti-N-methyl-D-aspartate receptor (anti-NMDAR) encephalitis is an important type of brain inflammation caused by autoantibody. As one of the primary agents responsible for respiratory tract infection, the human respiratory syncytial virus (hRSV) has also been reported to be capable of causing extrapulmonary diseases. Here, we first describe a case of anti-NMDAR encephalitis when hRSV was shown to be present in the cerebrospinal fluid.

**Case presentation:**

The child was noted to have ataxia and positive anti-NMDA receptors in the cerebrospinal fluid, diagnosed as anti-NMDA receptor encephalitis in combination with cranial MRI images. After high-dose hormone pulse therapy and medication, the disease improved, and he was discharged. However, a relapse occurred almost a year later, and the cranial MRI imaging showed progressive cerebellar atrophy. An hRSV strain from group B was detected in his cerebrospinal fluid, and the whole genome sequence was recovered using transcriptome sequencing.

**Conclusions:**

To our knowledge, this is the first report of hRSV being found in the cerebrospinal fluid of a patient with anti-NMDAR encephalitis. Even though more clinical records and experimental evidence are needed for validation, this work expands the types of diseases linked to hRSV and the likely cause of anti-NMDAR encephalitis.

**Supplementary Information:**

The online version contains supplementary material available at 10.1186/s12985-023-01997-1.

## Background

Encephalitis is a kind of inflammatory condition of the brain, resulting in substantial morbidity and mortality cases, especially in children [1]. The cause of encephalitis usually involves aspects of infectious agents or autoimmune conditions. In the past, even though many pathogens were uncovered for their association with encephalitis, it was widely accepted that autoimmune conditions contributed significantly to the etiology of encephalitis [2]. There are various antibodies to different types of antigens involved in autoimmune encephalitis, such as anti-Hu, ion channels, receptors, and other associated proteins [3, 4]. Anti-N-methyl-D-aspartate receptor (NMDAR) encephalitis, the most frequent kind of autoimmune encephalitis, manifests a wide variety of clinical symptoms, including short-term memory loss, reduced or changed state of consciousness, psychiatric symptoms, focal central nervous system (CNS) abnormalities, and new-onset seizures [4]. The etiology of anti-NMDAR encephalitis is complex and multifactorial, mainly involving tumors and viral infection [5–8]. In comparison to adults, because tumors are uncommon in children, the virus that induces this disease is most frequently documented in pediatric cases, particularly the herpes simplex virus [6, 7, 9]. Moreover, some other viral agents were also found to have a potential relationship with anti-NMDAR encephalitis [10, 11]. In this study, we reported an 12-year-old child with obvious clinical signs of ataxia who was eventually diagnosed with anti-NMDAR encephalitis. Furthermore, deep sequencing revealed the presence of a human respiratory syncytial virus (hRSV) strain from group B in the cerebrospinal fluid (CSF) of this case. Despite further experimental evidence being deserved, a primary clue to the role of the hRSV associated with anti-NMDAR encephalitis, such as the candidate trigger, is proposed.

## Case presentation

**Fig. 1:**
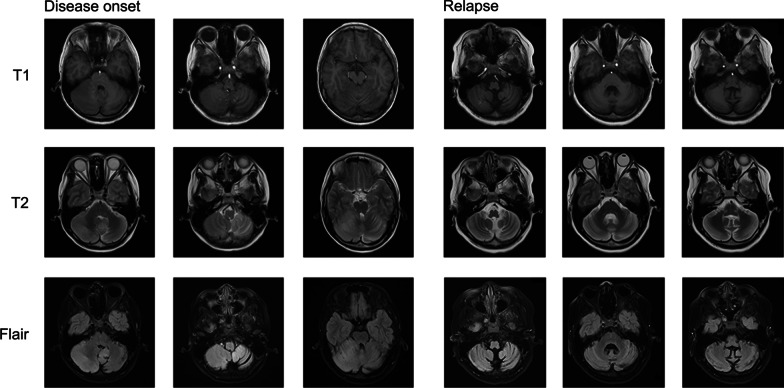
Cranial MRI images of the case in the phases of disease onset and relapse, including T1, T2, and FLAIR sequences

In November 2019, a boy aged 12 was admitted to the Wuxi Children’s hospital for intermittent dizziness, headaches for more than a month, and unstable walking for half a month. It is noted that the patient had a mild cough a week before the onset, and it resolved spontaneously without intervention. Furthermore, the boy has not received any vaccinations since he was 6 years old. Some neurological tests, such as the Oppenheim test, Brudzinski's sign, Kernig's sign, and Babinski's sign, yielded negative results, whereas the Tandem Gait and finger-to-nose test yielded positive results, particularly in the right hand. In addition, a spectrum of demyelinating diseases in the central nervous system, such as antibodies against aquaporin-4, myelin oligodendrocyte glycoprotein, and glial fibrillary acidic protein, were negative. The following autoimmune encephalitis relevant tests revealed that AMPA1/2 receptors, and GABABR in CSF were negative, but the NMDA-IgG was positive with a titer of 1:32 (Additional file 1: Fig. S1). Brain MRI revealed aberrant signals in both cerebellar hemispheres, indicating significant cerebellar atrophy (Fig. 1). Eventually, the boy was diagnosed with anti-NMDAR encephalitis. The detection of some tumor markers, including alpha-fetoprotein, carcinoembryonic antigen, carbohydrate antigen (CA) 125, and CA 19–9, was performed to rule out the possibility of tumors, and all were negative. Then, this patient was treated with pulse therapy of methylprednisolone sodium succinate (20 mg/kg, 3d) and later changed to prednisolone tablets (40 mg, po, qd). Finally, the boy's symptoms were relieved, and he was discharged from the hospital. Follow-up visits in January and May 2020 revealed that this child was in good health with no noticeable symptoms. However, the child was readmitted to the hospital in August 2020 with dysgraphia for 6 days. The neurological examination results were comparable to the last time, with the finger-to-nose test remaining positive, but the Tandem Gait was negative. The NMDAR-IgG titer was 1:10 in serum and 1:3.2 in CSF (Additional file 1: Figs. S2 and S3), and MRI imaging revealed worsened lesions in the cerebellar hemispheres (Fig. 1). Besides, the CSF oligoclonal IgG band test was performed, and the result was positive. The positive anti-NMDAR-IgG combined with imaging results led to the diagnosis of relapsing anti-NMDAR encephalitis. A similar therapeutic schedule was applied, and the boy was discharged after improving his symptoms. Later follow-ups revealed that the patient's symptoms were eased to a certain extent, but there was a recurrence. Additionally, his memory and learning skills were somewhat impaired. More details of the clinical features and test results can be found in the Additional file 2 and 3.

CSF transcriptome sequencing was performed in both episodes to help further interpret the etiology of this case. Before the examination, informed consent was obtained from the boy and his parents. Briefly, the total RNA of CSF was extracted using the RNeasy Micro Kit (Qiagen) following the manufacturer's instructions, from which the host rRNA was removed before the sequencing library construction. The library was then constructed using the Trio RNA-seq Kit (Nugen), and the meta-transcriptomic sequencing was performed on the Illumina hiseq X-ten platform as described previously [12]. The resulting raw reads were first trimmed to remove those of low quality and adapters using Trimmomatic software [13], and the reads of human origin were removed using the in-house script. The generated clean reads were then directly applied to the blast analysis against the nr database in NCBI to parse the taxonomic composition using BLAST + 2.12.0. Afterward, the reads of interest were mapped to the reference sequences to resolve the coverage using bowie2, and then de novo assembled using the Megahit program under default parameters [14]. Then, the assembled contigs were used as queries for the Blast analysis to confirm their taxonomic status. Furthermore, the MEGA program was used to align and build a maximum likelihood tree for the phylogenetic relationship with other reference strains [15].

**Fig. 2:**
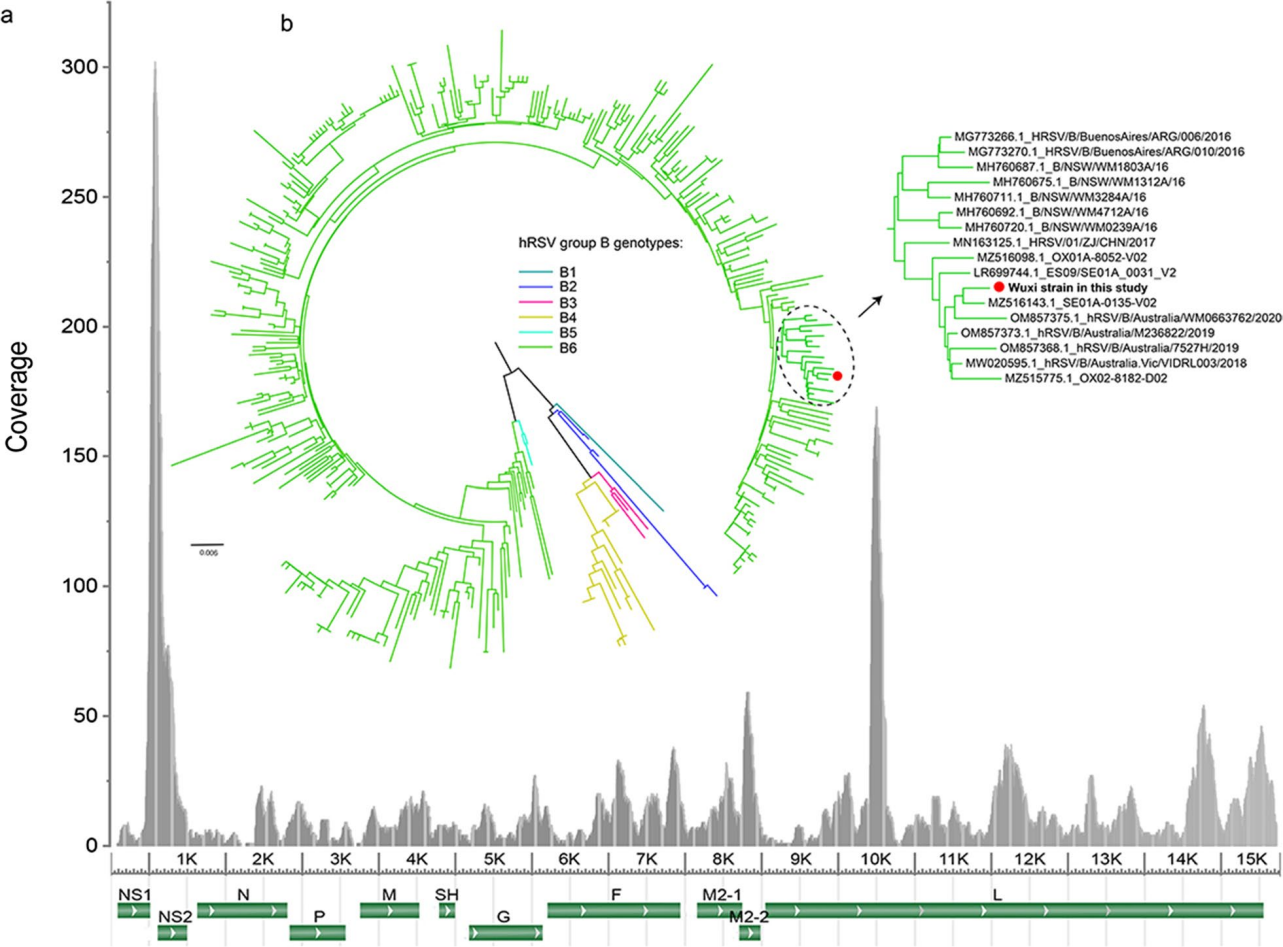
**a** The mapping analysis of the detected hRSV-associated reads. The reference genome was the hRSV group B strain SE01A-0135-V02. The genome organization of this strain is shown below; **b** the phylogenetical relationship of the detected Wuxi strain with other reference strains from various genotypes

For the sequencing in the first episode, there were 37,013,105 paired reads generated in total, among which the majority were assigned to Homo sapiens as expected. Based on the read blast analysis, those probably belonging to eukaryota, bacteria, and viruses accounted for 70.54%, 17.04%, and 7.61%, respectively, of the reads irrelevant to humans. Moreover, similar results were achieved from the blast analysis using assembled contigs as queries. Among these reads from putative exogenous agents, a total of 2459 can be mapped to hRSV type B (strain SE01A-0135-V02) with coverage of 98.5% of its genome sequence (accession no. MZ516143). In particular, most reads concentrated on the location of genes NS1, NS2, and L (Fig. 2a). RT-PCR and sanger sequencing were used to fill in the gaps in the assembled contigs and confirm the genome sequences. Finally, with a total of 15,184 bases, the sequence of the whole genome was recovered, except for part of the 3' UTR region. Furthermore, it had been deposited in the Genbank (accession no. ON630422). Based on the sequence analysis, the acquired genome sequence shared 99.69% nucleotide identity with SE01A-0135-V02. Afterward, using the ML tree based on the G gene, the phylogenetic relationship of the Wuxi strain with other genotypes of hRSV group B was disentangled. The results indicated that this strain grouped with SE01A-0135-V02 inside B6, the most prevalent hRSV genotype in group B worldwide (Fig. 2b). The remaining reads were dispersed to other taxonomic groups with low read numbers or short assembled contigs. The second sequencing resulted in 34,609,924 paired reads, and except for reads from human beings, no reads associated with agents were confirmed relevant to human disease conditions. Due to the absence of the antibody or antigen tests of hRSV at the time of onset, we conducted the test of serum IgG to the hRSV after the CSF sequencing using the Human RSV-IgG ELISA Kit (SHUANGYING BIOLOGICAL), and the result was positive (Additional file 1: Fig. S4).

## Discussion and conclusions

Whether in the first episode or relapse, the first noticeable symptom of the illness in this boy is abnormal movements and autonomic instability. The comparison of the MRI imaging findings of the two phases demonstrated progressive cerebellar atrophy. Although this contrasts with the onset of typical psychiatric symptoms seen in most anti-NMDAR encephalitis cases, neurologic symptoms, such as abnormal movements or seizures, are considered significant onset symptoms in children [16–18]. Besides, oligoclonal IgG bands were identified, which were also observed in some other children's cases, although the identical syndrome of multiple sclerosis was not observed in this case [19]. Regarding imaging results, the current patient showed evident cerebellar atrophy, which differed from earlier reported hRSV-associated or anti-NMDAR encephalitis cases [20, 21]. This case's clinical syndromes were atypical to some extent compared to other reported anti-NMDAR encephalitis cases.

Human orthopneumovirus, commonly known as a human respiratory syncytial virus (hRSV), is a member of the genus Orthopneumovirus in the family *Pneumoviridae*, a group of enveloped negative-strand RNA viruses [22]. There are two accepted subtypes, A and B, within hRSVs, followed by some unclassified strains [23]. The hRSV was primarily identified as an essential agent for respiratory tract infection in bronchiolitis and pneumonia [24]. Beyond this, this virus was also found to cause extrapulmonary diseases such as myocarditis, hyponatremia, and encephalitis [21, 25, 26]. Notably, a single hRSV-infected pediatric case with limbic encephalitis was also reported as positive for NMDAR antibodies (Additional file 3). Nevertheless, the virus was solely detected in the nasopharyngeal aspirate while negative in the CSF [27]. Otherwise, the presence of this virus in the CSF of some cases with hRSV-associated neurological abnormalities has been reported in some studies. Despite the lack of laboratory evidence yet confirming that the hRSV is neuroinvasive, some in vitro investigations have revealed that this virus can infiltrate the central nervous system and infect resident cells, which might explain why the virus can cause neurological disorders in people[28–30]. Although the mechanisms driving neurological complications caused by hRSV infection are obscure, it is proposed that hRSV-associated encephalopathies can be divided into four types, among which the cytokine storm type is involved [31]. Definitely, there has been sufficient clinical evidence of an altered profile of cytokines, such as IL-6, IL8, CCL2, and CCL4, in the CSF of hRSV-infected cases [32]. Interestingly, the altered cytokine levels in the CSF of anti-NMDAR encephalitis cases were also proposed as diagnosis markers [33]. Whether autoimmune encephalitis represents a novel form of hRSV-infection-related encephalopathy or merely a symptom of the brain's disturbed immunologic balance, hRSV is likely to play an important role in the condition in this case. Nonetheless, the etiology of the relapse, whether due to altered immunological homeostasis in the brain or an unknown trigger, requires more exploration. Considering this disease is prone to recurrence, the occurrence of relapsing encephalitis following the hRSV infection was a high possibility.

Taken together, we first identified the existence of hRSV group B in the CSF of a child with anti-NMDAR encephalitis. The study is limited by the dearth of solid immunological evidence (such as IgM from the onset of symptoms), as IgG merely confirms that the patient was infected with the virus. Even if further clinical records and experimental data on pathogenesis and more similar cases are required for validation, this work provides a starting point for future research on hRSV-associated anti-NMDAR encephalitis.

## Supplementary Information

**Additional file 1.** Supplementary figures.

**Additional file 2.** Detailed clinical characteristics and prognosis of the patient.

**Additional file 3. Supplementary Table 1.** Results of some clinical tests during hospitalization.

## Data Availability

The datasets supporting the conclusions of this article are included within the article.

## Abbreviations

AMPA: α-Amino-3-hydroxy-5-methyl-4-isoxazolepropionic acid
anti-NMDAR: Anti-N-methyl-D-aspartate receptor
CA: Carbohydrate antigen
CCL: Chemokine (C–C motif) ligand
CNS: Central nervous system
CSF: Cerebrospinal fluid
ELISA: Enzyme-linked immunosorbent assay
GABABR: G-protein coupled receptors for gamma-aminobutyric acid
hRSV: Human respiratory syncytial virus
IL: Interleukin
MRI: Magnetic resonance imaging
po: Orally
qd: Once a day
RT-PCR: Reverse transcription polymerase chain reaction
UTR: Untranslated region
