# Supplementary material for: The presence of human respiratory syncytial virus in the cerebrospinal fluid of a child with Anti-N-methyl-D-aspartate receptor encephalitis of unknown trigger
Source: Virol J. 2023 Feb 24;20:34. doi: 10.1186/s12985-023-01997-1 (PMC9951452; doi:10.1186/s12985-023-01997-1)
Supplement: Supplementary file 1 — Additional file 1. Supplementary figures. [file 12985_2023_1997_MOESM1_ESM.docx]

Supplementary figures.


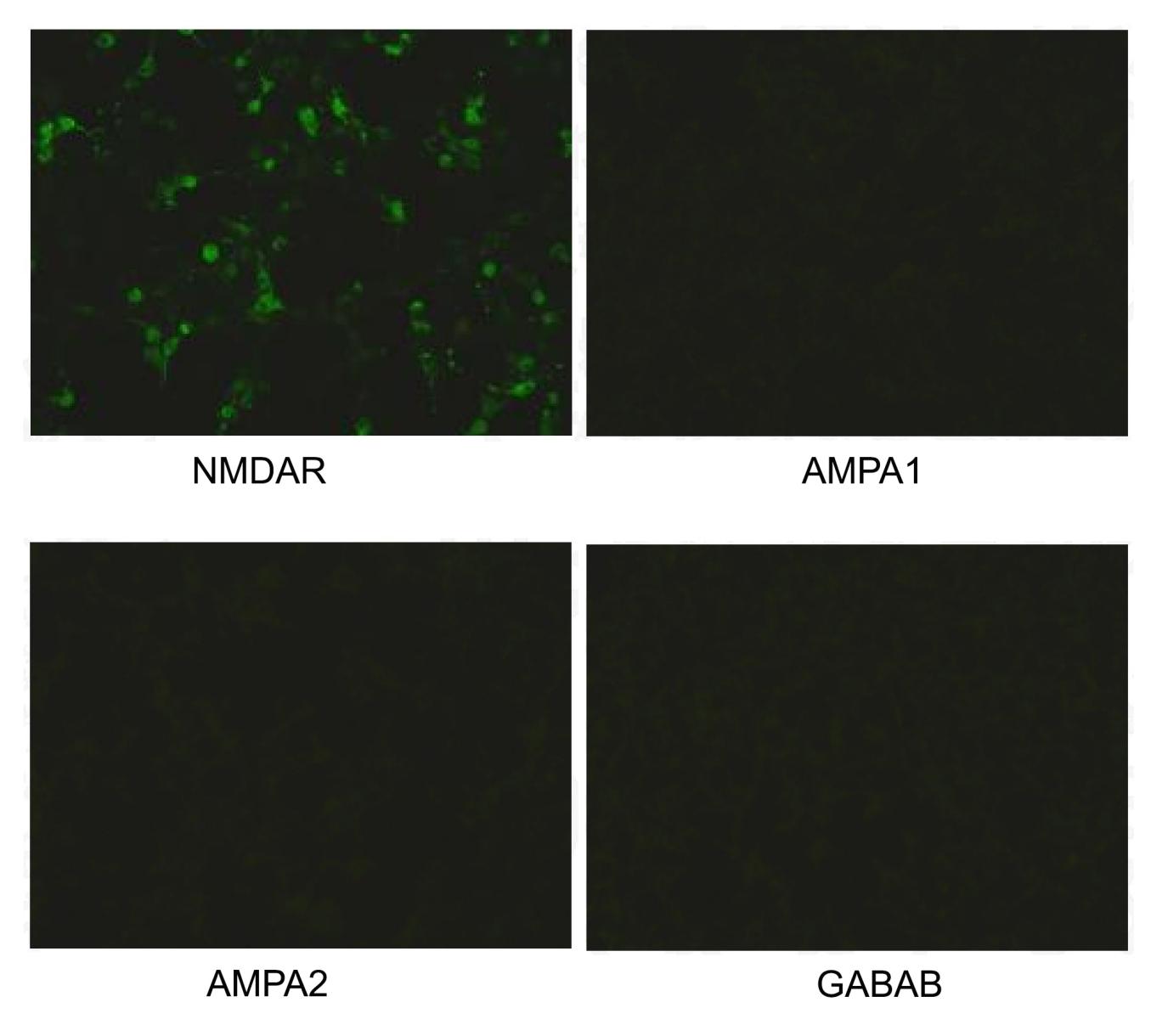


Figure S1: The results of some autoimmune encephalitis associated antibodies detection in CSF of the patients during his first hospitalization. The detection was using indirect immunofluorescence test (IIFT) conducted by V-medical laboratory (http://www.ivydx.com/xiangmu001.html). These figures are the IIFT imaging results of various transfected cells (the same below). The NMDAR was positive with titer of 1:32.


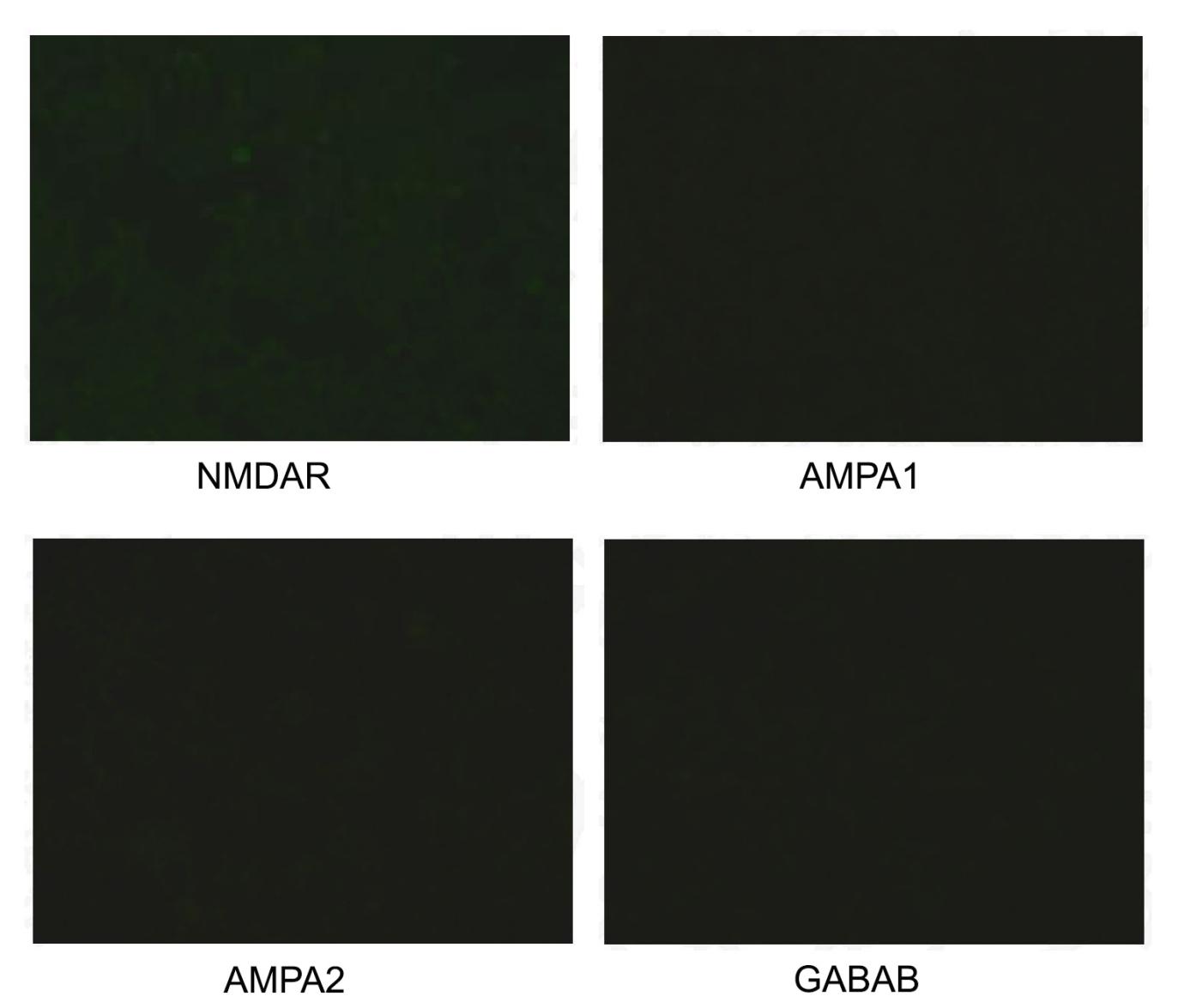


Figure S2: The results of some autoimmune encephalitis associated antibodies detection in serum of the patients during his second hospitalization. The NMDAR was positive with titer of 1:10.


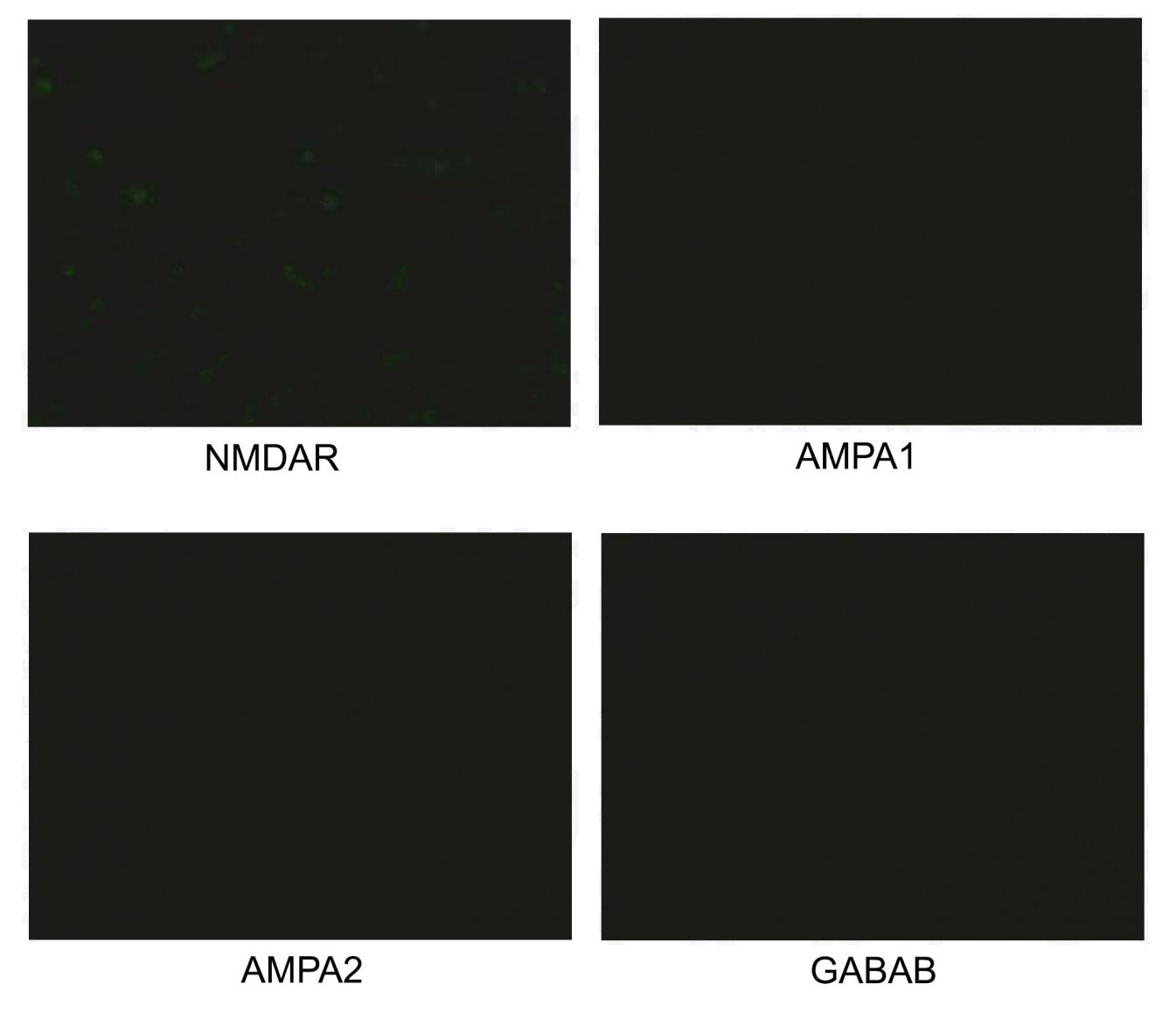


Figure S3: The results of some autoimmune encephalitis associated antibodies detection in CSF of the patients during his second hospitalization. The NMDAR was positive with titer of 1:3.2.


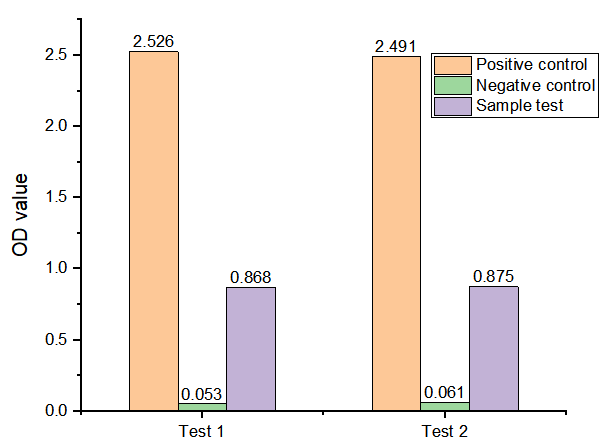


Figure S4. Test results of serum IgG to the hRSV. The test was conducted using the Human RSV-IgG ELISA Kit instruction (SHUANGYING BIOLOGICAL). Test validity: the average of Positive control well≥1.00 (present study: 2.509); the average of Negative control well ≤0.15 (present study:0.057); Calculate Critical (CUT OFF): Critical= the average of Negative control well + 0.15 (present study: 0.207); Negative Result: sample OD< Calculate Critical (CUT OFF) is Negative; Positive Result: sample OD≥ Calculate Critical (CUT OFF) is Positive (present study: 0.872). According to the instruction of the kit, the result was positive.
