# Supplementary material for: The presence of human respiratory syncytial virus in the cerebrospinal fluid of a child with Anti-N-methyl-D-aspartate receptor encephalitis of unknown trigger
Source: Virol J. 2023 Feb 24;20:34. doi: 10.1186/s12985-023-01997-1 (PMC9951452; doi:10.1186/s12985-023-01997-1)
Supplement: Supplementary file 2 — Additional file 2. Detailed clinical characteristics and prognosis of the patient. [file 12985_2023_1997_MOESM2_ESM.docx]

Detailed clinical characteristics and prognosis of the patient.

**First time admitted (2019-11)**

The boy was 12 years and 1 month old at the time of his initial onset, with intermittent dizziness and headaches for more than a month and symptoms associated with ataxia, such as "walking instability."

**Basic condition of the patient on admission is as follows:**

Body temperature: 37.1℃,

Pulse rate: 82 beats/min,

Respiration rate: 20 breaths/min,

Blood pressure: 115/62mmHg,

Weight: 34kg,

The boy's consciousness before being hospitalized was good, and so was his mental status. The patient's pupils were equal in size and round on both sides, 4 mm in diameter, responsive to light, and without nystagmus. His pharynx was not congested, the tonsils on both sides were not enlarged, cervical resistance was evident, and the breath sounds in both lungs were clear. Besides, the heart rhythm, heart sound, muscle strength and muscle tone of the limbs, and the Achilles tendon reflex were normal. Nevertheless, the knee-tendon reflex and biceps reflex were present.

**The results of some neurological tests on admission:**

Oppenheim test: Negative

Goldenhar syndrome: Negative

Brudzinski's sign: Negative

Kernig's sign: Negative

Babinski sign: Negative

Finger-to-nose test: positive

Tandem Gait (Heel-to-Toe): Positive

The MRI revealed abnormal signals in bilateral cerebellar hemispheres, while the test results of some other examinations, such as B ultrasound of the carotid artery, electrocardiograph, and head CT, were all in the normal range.

**Diagnosis and treatment process after admission to the hospital**

The patient's condition worsened after admission, and mannitol was used to reduce intracranial pressure. A further test of cranial MRI enhancement was performed, thus ruling out the possibility of an intracranial occupying lesion. The patients were then subjected to lumbar puncture and cerebrospinal fluid examination. The cerebrospinal fluid (CSF) was taken for autoimmune encephalitis associated and oligoclonal antibodies, while the blood was tested for oligoclonal antibodies and those against such as AQP4 and MOG. The remaining CSF was collected to conduct the meta-transcriptomics analysis.

The increased leucocyte count in the CSF was observed (60*10^6^/L), and combined with the MRI result, encephalitis resulting from an autoimmune disorder was considered. Accordingly, methylprednisolone sodium succinate 20 mg q12h was given as anti-inflammatory and immunosuppressive treatment, and oral calcium and potassium supplementation. The antibody test results in CSF revealed that anti-NMDA IgG was positive with a titer of 1:32, which is further proof of the diagnosis of anti-NMDAR encephalitis. Then the high-dose methylprednisolone (20mg/kg*3d) was used and replaced by prednisone tablets (40mg, po, qd) afterward.

After treatment, the child significantly improved the symptoms of ataxia, particularly in walking gait. Due to the improvement, the child was discharged with medication (prednisone tablets, 40mg, po, qd; Vitamin D calcium, 1 tablet, po, qd; Potassium Chloride Sustained-Release Tablets, 0.5g, po, bid) at the request of his parents.

**Subsequent visit 1 (2020-01)**

The patient is in good health with no recurrence of related neurological symptoms

**Subsequent visit 2 (2020-05)**

The patient is in good health with no recurrence of related neurological symptoms

**Relapse (2020-08)**

Nine months after being discharged from the hospital, the boy was admitted for a second time because of writing difficulties lasting six days.

**Basic condition of the patient on admission is as follows:**

Body temperature: 36.7℃,

Pulse rate: 85 beats/min,

Respiration rate: 20 breaths/min,

Blood pressure: 108/65mmHg,

Weight: 45kg,

The boy's consciousness before being hospitalized was good, and so was his mental status. The patient's pupils were equal in size and round on both sides, 4 mm in diameter, responsive to light, and without nystagmus. His pharynx was not congested, the tonsils on both sides were not enlarged, cervical resistance was evident, and the breath sounds in both lungs were clear. Besides, the heart rhythm, heart sound, muscle strength and muscle tone of the limbs, and the Achilles tendon reflex were normal. However, the biceps reflex was present.

**The results of some neurological tests on admission:**

Oppenheim test: Negative

Goldenhar syndrome: Negative

Brudzinski's sign: Negative

Kernig's sign: Negative

Babinski sign: Negative

Finger-to-nose test: positive

Tandem Gait (Heel-to-Toe): Negative

**Diagnosis and treatment process after admission to the hospital**

After admission, the child was given vitamin B6 tablets orally. On the second day of admission, the lumbar puncture and routine CSF exam were done, as well as tests for autoimmune encephalitis antibodies in the serum and CSF and MOG antibodies in the serum. Based on the previous experience and the relevant test results, a treatment similar to the previous phase was applied to this child (sodium methylprednisolone succinate as an anti-inflammatory drip and potassium and calcium supplementation). There were no mental or behavioral problems, convulsive seizures, speech or movement problems, and vision loss. After 9 days, this patient's symptoms resolved, and he was discharged from the hospital.

After discharge from the hospital, he remained on medication, prednisone tablets: 60 mg, po, qd, maintained for 2 months, with a slow reduction after 2 months, for a total course of about 10 months.

**Subsequent visit 3 (2020-10)**

The hormone treatment is still in use, and after the hand trembling when writing has improved, it has recurred.

**Subsequent visit 4 (2021-8)**

The child's hormone therapy has been stopped for a month, and he is in good mental condition. The speech is slow, and the hand trembles when writing has improved. There is some memory loss, which affects academic performance.
