# Supplementary material for: The presence of human respiratory syncytial virus in the cerebrospinal fluid of a child with Anti-N-methyl-D-aspartate receptor encephalitis of unknown trigger
Source: Virol J. 2023 Feb 24;20:34. doi: 10.1186/s12985-023-01997-1 (PMC9951452; doi:10.1186/s12985-023-01997-1)
Supplement: Supplementary file 3 — Additional file 3. Supplementary Table 1. Results of some clinical tests during hospitalization. [file 12985_2023_1997_MOESM3_ESM.docx]

**Supplementary Table 1. Results of some clinical tests during hospitalization**

| Date | Tests | Results | Normal range |
| --- | --- | --- | --- |
| 2019-11-5 | **Blood cell analysis** |  |  |
|  | White blood cell count | 7. 89*10^9^/L | 4-10*10^9^/L |
|  | Neutrophil ratio | 71.2% | 50-70% |
|  | Lymphocyte ratio | 20. 0 % | 20-40% |
|  | Hemoglobin | 132g/L | 110-140g/L |
|  | Blood platelet count | 200*10^9^/L | 125-350*10^9^/L |
|  | C-reactive protein | 1.9mg/L | 0-8mg/L |
|  |  |  |  |
| 2019-11-16 | **Blood cell analysis** |  |  |
|  | White blood cell count | 11. 08*10^9^/L | 4-10*10^9^/L |
|  | Neutrophil ratio | 72.4% | 50-70% |
|  | Lymphocyte ratio | 14.7% | 20-40% |
|  | Hemoglobin | 151g/L | 110-140g/L |
|  | Blood platelet count | 291*10^9^/L | 125-350*10^9^/L |
|  | C-reactive protein | 1.9mg/L | 0-8mg/L |
|  |  |  |  |
| 2019-11-5 | Creatinine | 33. 1 umol/L | 71-133umol/L |
| 2019-11-5 | Lactic acid | 1.3mmol/L | 0.7-2.1mmol/L |
| 2019-11-5 | Blood ammonia | 31. 73μmol/L | 9-33μmol/L |
|  |  |  |  |
| 2019-11-11 | CSF IgG index | 0.99 | ≤0.7 |
|  |  |  |  |
|  | **Liver function** |  |  |
| 2019-11-16 | TBil(total bilirubin) | 4.9μmol/L | 2.0-20.0 |
|  | lactic dehydrogenase | 479U/L | 109-245 |
|  |  |  |  |
|  | **Tumor markers** |  |  |
| 2019-11-6 | AFP(alpha-fetoprotein) | 1.09 ng/ml | ＜10 |
|  | carcinoembryonic antigen | 1.32 ng/ml | ＜5 |
|  | carbohydrate antigen CA125 | 13. 40U/ml | ＜35 |
|  | carbohydrate antigen CA19-9 | ＜0.80U/ml | ＜35 |
|  |  |  |  |
| 2019-11-8 | **Routine CSF** |  |  |
|  | Color | colorless | colorless |
|  | transparency | transparent | transparent |
|  | Protein qualitative | Negative | Negative |
|  | RBC（red blood cell） | 20*10^6^/L | 0-1*10^6^/L |
|  | White blood cell count | 60*10^6^/L | 0-8*10^6^/L |
|  | neutrophil cells | 5% | 0-6% |
|  | lymphocyte | 95% |  |
|  |  |  |  |
| 2019-11-8 | **Biochemical analysis of CSF** |  |  |
|  | Chlorine | 121. 6 mmol/L | 120-130 |
|  | Glucose | 3. 45 mmol/L | 2.2-3.9 |
|  | Protein quantification | 0. 42 g/L | 0.12-0.6 |
|  |  |  |  |
| 2019-11-8 | Bacterial culture and drug-sensitive test of CSF | Negative | Negative |
|  | T-cell detection for tuberculosis infection | Negative | Negative |
|  |  |  |  |
| 2019-11-7 | Antithyroglobulin antibody | 6. 40% | 0-30 |
|  | Thyrotropin receptor antibody | 12. 20U/L | ＜14 |
|  | Anti-thyroid microsomal antibody | 6.90% | 0-20 |
|  | anti-thyroid peroxidase antibody | 1. 33IU/ml | 0-100 |
|  |  |  |  |
|  | **Thyroid** |  |  |
| 2019-11-6 | triiodothyronine | 1.81 nmol/L | 1.3-3.1 |
|  | thyroxine | 102. 20 nmol/L | 66.0-181.0 |
|  | thyroid stimulating hormone（TSH） | 1.60 uIU/ml | 0.27-4.20 |
|  | free triiodothyronine | 5.08pmol/L | 3.1-6.8 |
|  | free thyroxine | 16. 28 pmol/L | 12.0-22.0 |
|  |  |  |  |
| 2020-8-21 | **Blood cell analysis** |  |  |
|  | White blood cell count | 4.52*10^9^/L | 4-10*10^9^/L |
|  | Neutrophil ratio | 49.1% | 50-70% |
|  | Lymphocyte ratio | 40.9 % | 20-40% |
|  | Hemoglobin | 134g/L | 110-140g/L |
|  | Blood platelet count | 218*10^9^/L | 125-350*10^9^/L |
|  | C-reactive protein | ＜0.5mg/L | 0-8mg/L |
|  |  |  |  |
| 2020-8-22 | **Routine CSF** |  |  |
|  | Color | colorless | colorless |
|  | transparency | transparent | transparent |
|  | Protein qualitative | Negative | Negative |
|  | RBC（red blood cell） | 100*10^6^/L | 0-1*10^6^/L |
|  | White blood cell count | 32*10^6^/L | 0-8*10^6^/L |
|  | neutrophil cells | 10% | 0-6% |
|  | lymphocyte | 90% |  |
